# Quality and quantity of construction and demolition waste in Tehran

**DOI:** 10.1186/s40201-017-0276-0

**Published:** 2017-06-20

**Authors:** Alireza Asgari, Tahereh Ghorbanian, Nader Yousefi, Dariush Dadashzadeh, Fatemeh Khalili, Amin Bagheri, Mehdi Raei, Amir Hossein Mahvi

**Affiliations:** 1 Department of Environmental Health Engineering, School of Public Health, Tehran University of Medical Sciences, Tehran, Iran; 2 Center for Solid Waste Research, Institute for Environmental Research, Tehran University of Medical Science, Tehran, Iran; 3 Environmental and Occupational Health Center, Ministry of Health, Tehran, Iran; 4 Department of Basic Sciences, School of medicine, Qom University of Medical Sciences, Qom, Iran; 5 National Institute of Health Research, Tehran University of Medical Sciences, Tehran, Iran

**Keywords:** Construction and demolition waste, Waste minimization, Recycling, Reuse, Waste management

## Abstract

**Background:**

In recent years the generation rate of construction and demolition waste (C&D) has significantly augmented. The aim of this study was to assessed the quality and quantity of construction and demolition waste in Tehran (capital of Iran).

**Methods:**

Questionnaire methods were used for estimating the amount of generated C&D wastes national statistical data and typical waste generation data. In order to defining the composition of C&D waste, trucks were randomly selected and their wastes were separated and weighted.

**Results:**

According to obtained results, about 82,646,051 m^3^ of C&D waste (average 16,529,210 m^3^ per year) were generated during 2011 to 2016 which only about 26% of them has been recycled. Mixing sand and cement, concrete, broken bricks and soil have the highest amount of the composition of C&D waste in Tehran that was 30, 19, 18 and 11%, respectively. Based on the results, about 2,784,158 t of the waste will generate in 2025 and this is approximately 122% higher than wastes generate in 2016. Based on MAPSA’s data, 360 teams of personnel cruise and control the illegal disposals, but due to the expansion of Tehran this number of teams is inadequate and can’t be effective in controlling the situation.

**Conclusion:**

In general, the overall condition of C&D waste management in Tehran seems undesirable and needs to be updated based on the experience of successful countries in this field.

## Background

Construction and demolition [1] debris generates from several activities like the renovation, construction and demolition of buildings, bridges, streets, public residents, roads and other structures. Although all materials that was categorized as a C&D debris depend on the regulatory body [2–6], the typical components of construction and demolition (C&D) debris are drywall, concrete, wood, asphalt, soil, metal and packaging materials such as paper and plastic [7]. When new structures like residential and nonresidential buildings, public works projects are constructed and/or existing structures are demolished or renovated (including deconstruction activities), these types of solid wastes are generated [4]. While solid wastes that are classified as the C&D materials vary from place to place, C&D materials can include the vegetation materials that are generates when a new site is constructed as well as land clearing debris [8]. In general, C&D waste is a concern at all over the world because of the quick growth of urbanization and a notable number of impermissible dumps [9].

C&D waste sources have generally produced from certain sources. According to Graham and Smithers opinion, the main sources of construction and demolition waste could be different activities during various projects. Potential sources are design phase, procurement phase like shipping and ordering error, handling of the materials phase such as inappropriate storage and improper handling activities on and off site, operation phase like equipment error, accidents and human faults and weather, residual (surplus foods, non-consumables) and other activities like vandals and clients actions [10].

The C&D include a notable component of solid waste streams (20–30% and sometimes more than 50% of MSWs) [11, 12]. According to the studies, construction wastes are included about 35% of municipal solid wastes in developed countries and 50% in developing countries which are a major amount of MSWs [13]. C&D materials are generated from the construction industry of Canada is approximately 27% of the total municipal solid waste (MSWs) that discharged into the landfills [12], about 30% (based on the volume) of the total materials disposed in the US landfills [14] and about half of the total waste in New Zealand are C&D wastes [15]. In addition, 12% of California’s landfill space are occupied by C&D waste [4].

The most parts of the total world’s material and resources (32%) that include 12% of water body and resources and 40% of energy sources are used through construction activities. Furthermore, construction is consumed about 25% of virgin wood and 40% of raw materials draw out from the earth. C&D waste cause adverse or negative effects on the environment (water, soil and air pollution as well as negative impacts on the fauna and flora), social issues as well as public health (aesthetic aspect, health hazards, reproduction of pests and rodents, working and human safety), economy (fuel consumption through transportation and loss of raw materials and primary resources) [16].

Some studies were conducted in different countries about construction and demolition waste management. The improvement of the Statutory Framework for this kind of waste has been reviewed in Germany and Australia. According to this study results, waste management activities and consequences may vary between areas and countries. For example, in Australia, handling of C&D waste was gradually amended during the previous years, nevetheless the quantity of generated C&D waste increased annually, as waste avoidance and minimization has a little advancement. In contrast, in Germany, there are some successful plan for waste minimization and management whenever the waste management and recycling program performed in Germany is one of the prospering experience in the world. Nonetheless, demolition work is the most waste recycled from solid waste stream [9]. In another study, the C&D waste generation and management have been estimated in Thailand during 2002 to 2005. According to this study, about 1.1 million tons per year of C&D wastes were generated in Thailand. This includes less than 10% of the total waste disposed in landfills as well as open dumpsites. Also, according to the that research, wood, concrete, tiles and bricks and steel reinforcement were the most part of this type of solid waste in Thailand [16]. Generally, the most common reason for understanding the precise amount of C&D wastes generated or recovered in the MSW streams is determining a comprehensive materials recovery programs. Diverting and recycling of C&D wastes from solid waste stream can lead to save the natural resources, decrease the emissions of greenhouse gas, reduce the landfill space requirement and save money [8].

The main limitations in this study were the lack and/or unavailability to C&D waste data in Tehran. Therefore, design of plan for future requirements for C&D waste management is really hard and it is essential that municipal of Tehran creates a statistical database about C&D waste.

The aim of this study was to specify the quality and quantity of C&D wastes and survey the management strategy of these materials in Tehran as capital of Iran and one of the biggest metropolises of Iran and Asia.

## Methods

### Studied area

This cross-sectional study has been conducted in Tehran as capital of Iran since 2011 to 2016. The population of Tehran was 8,429,807 according to the recent national census in 2012. Tehran is also capital and largest city of Iran and the 21st largest city in the world. Its altitude is between 1050 and 1800 m above sea level. Average annual temperature and relative humidity in Tehran is about 15 °C, 40%. Average annual precipitation of Tehran is estimated about 242 mm.

### Sampling method

**Fig. 1:**
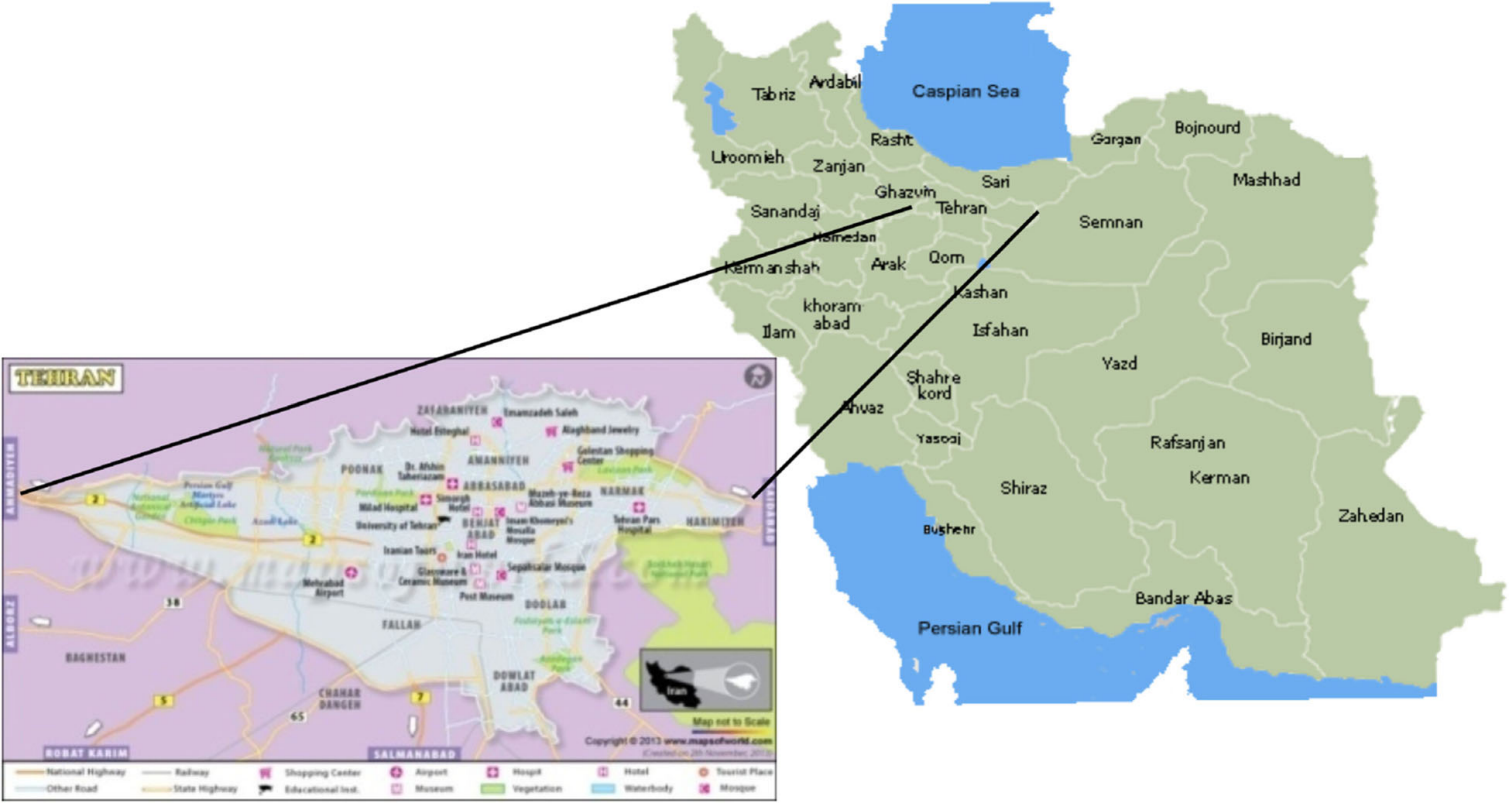
Location of the studied area in Iran and Tehran

The methodology used in this study for calculating the amount of generated C&D waste was taken from national statistical data and waste generation data extracted from questionnaires. The amount of materials results from building construction, demolition and renovation activity were gotten the Abdal Industrial Projects Management (MAPSA) and national statistical data. Situation of Tehran in Iran and sampling point locations in Tehran are shown in Fig. 1.

In general, there are two ways to ascertain the amount of C&D waste. The first way is measuring the amount of C&D waste that entered to the landfills.

One of the main challenging task for the C&D waste management determine the direction for quantities and composition of C&D waste, because there are no oblige for recording the quality and quantity of the generated waste. As a result, number and area of construction license and the type of building activity were used to find the amount of generated construction waste. In this way, the amount of generated waste is defining using proper conversion ratio. To reach this goal, questionnaires were designed to determine the conversion ratio. Based on data obtained from five demolition contractors (Table 1) and also previous studies [17] the conversion ratio was determined 1.75.

**Table 1:** Conversion ratio of surface area to weight

Type of structures	Demolition contractors				
	1	2	3	4	5
Concrete structure	1.9	1.8	1.75	1.6	1.7
Steel structure	2	1.6	1.5	1.4	1.5
Masonry structures	2.5	1.6	1.8	1.6	1.7
Average	1.75				

Generated waste = Demolished area [18] * 1.75 (Conversion ratio)

In other way, the amount of C&D generation was detected as the average area of each building, the number of assigned building license and the material weights used for a typical and certain building type based on unit area. In addition, all materials which are physically in the building were considered as a results of demolition activities, and 10% of material which physically is in the building was considered as a construction waste. So the amount of generated C&D waste can be evaluated by the number and area of destroyed constructions. But unfortunately information about the number of construction and Demolition licenses did not exist in Tehran. In this way the amount of construction and maintenance of infrastructure should be considered.

The composition of C&D waste in Abali were defined by 15 workers. Some trucks were selected randomly and their wastes were separated and different materials were weighted.

To achieve the future status of C&D waste and its management according to the information during past few years obtained from MAPSA, WinPepi version 9.4 was used that calculated based on nonparametric regression analysis of time series data.

## Results and discussion

### C&D waste generation in Tehran

Based on the results obtained from MAPSA, the generated C&D waste in Tehran during the past 5 years was about 82,646,051 m^3^ (average 16,529,210 m^3^ per year) which only about 26% has been recycled in Rigsazan factory (Table 2). The amount of generated waste in Thailand was about 7,256,565 m^3^/y that is lower than the generated C&D waste in Tehran [16]. This value is so low according to population of Thailand (67 million). It seems that in this study did not consider some portions of C&D waste such as solid waste generated from any type of construction and demolition, operation and maintenance of the infrastructure (such as highways, bridges) that estimated by other studies. Generated C&D waste (ton per capita) in different countries were shown in Table 3. Based on the results of this study, C&D waste generation in Tehran has nearly similar condition to Denmark, Finland and Germany but recycling rate in Tehran is such lower than these countries. The range of generation per capita in these countries were between 0.04 and 5.9 t per capita in Latvia and Luxembourg, respectively. Luxembourg, France, Denmark and Finland had the high level of C&D waste generation and Lithuania, Poland, Bulgaria, Hungary, Greece and Slovakia had very low quantity.

**Table 2:** Construction and maintenance of infrastructure waste in Tehran

Laminating, cutting and manual asphalt (m^2^)	Creek and curb (m)	Pavement (m^2^)	Bridge (m)	Tunnel (m)	Stabilization, such as separation wall (m^2^)	Parks (m^2^)	Digging and wells (m^3^)
5,909,774	2,242,456	1,112,491	652,038	225,200	617,334	3,293,286	1,317,097

**Table 3:** Comparison between generated C&D waste in Tehran and other countries [20]

Country	C&D Waste arising (tones/capita)	Waste factor (1000 t/million € added value)
Tehran (Capital of Iran)	3.2	N/A
Austria	0.81	0.46
Belgium	1.06	0.955
Bulgaria	0.39	4.53
Cyprus	0.58	0.545
Czech Republic	1.44	4.037
Denmark	3.99	0.578
Estonia	1.12	4.144
Finland	3.99	3.239
France	5.5	5.016
Germany	2.33	2.406
Greece	0.37	0.344
Hungary	0.43	1.629
Ireland	2.74	1.312
Italy	0.8	0.778
Latvia	0.04	0.118
Lithuania	0.1	0.343
Luxembourg	5.9	N/A
Malta	1.95	N/A
Netherlands	1.47	1.264
Norway	0.7	0.194
Poland	0.11	0.41
Portugal	1.09	1.574
Romania	N/A	0.02
Slovakia	0.26	1.047
Slovenia	N/A	1.261
Spain	0.74	0.525
Sweden	1.14	1.029
United Kingdom	1.66	1.14
EU	1.74	N/A

The geographical variations of these countries cannot be considered to present actual increasing of the C&D waste. Differences in definitions and reporting mechanisms and the various levels of report and control of C&D waste are the main reasons for these discrepancies. In addition, another issue for determining the exact amount of C&D waste generated in the urban area is the quality of the available data [19].

Based on data obtained from MAPSA, the total amount of this type of waste was 15,369,686 m^3^ in Tehran. Most of the waste were generated in construction of bridges and tunnels. The amount of each construction and maintenance of infrastructure in Tehran is shown in Table 2.

A study in Germany showed that the main part of total C&D generated in 2002 is excavation materials (65.9%), demolition waste from building (24.3%), demolition waste resulted in road (7.8%) and finally construction site waste (2.0%) [20]. So the volume of this kind of waste is noticeable and should be considered the best technology and plan for managing these materials.

Generally, the amount of reusable or recyclable C&D waste is 50–80% [21]. Based on previous studies, the percentage of recycling is more than 80% in Denmark, in Australia 70–90%, and 30–50% in Germany, Finland, Italy, Netherlands and Ireland, and 10% in Luxembourg [3].

Recycling has a lot of economic and environmental benefits such as prolonging the life of landfill sites, reducing the use of energy and resource requirements, minimizing transport needs, creating job opportunities, increasing the income and etc.

Some programs were planned to recycle the C&D wastes in Tehran but none of them has been carried out.

The country’s C&D waste analysis presented a raising trend of generation in parallel with the improvement of the economy, rapid population growth especially in urban area [16]. In some other countries this correlation has been observed, too [16].

**Fig. 2:**
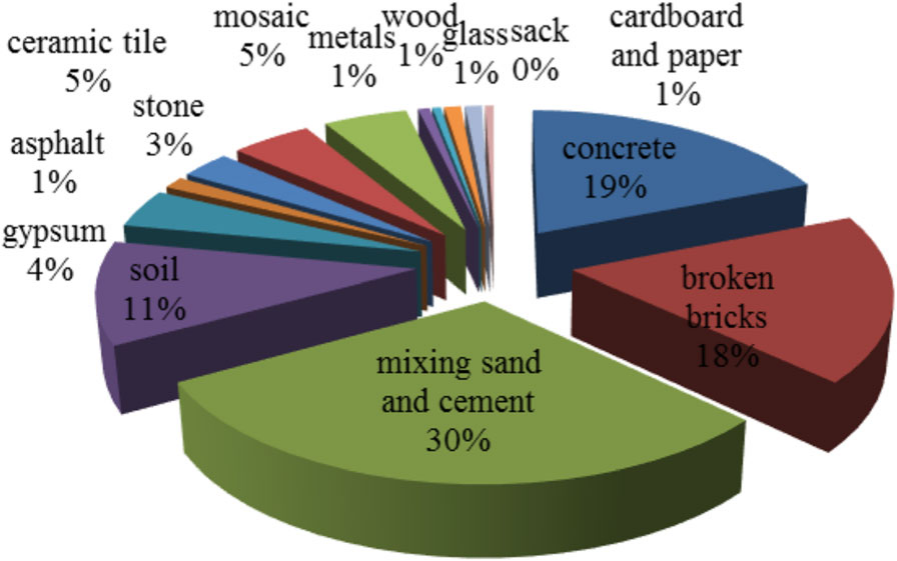
The composition of C&D waste in Tehran

The composition of C&D waste was determined (Fig. 2) and compared with some other cities in Iran (Table 4).

**Table 4:** Composition of C&D waste in Tehran and some cities of Iran [17, 32, 33]

Materials	Tehran (%)	Kermanshah (%)	Mashhad (%)	Shahrood (%)
Concrete	19	17	19.3	98.3
Broken bricks	18	19	13.8	
Mixing sand and cement	30	29.3	47.8	
Soil	11	12.5		
Gypsum	4.20	4.4	16.5	
Asphalt	1.30	2.1	-	
Stone	3	3.8	1.8	
Ceramic tile	4.80	3.7	0.4	1.7
Mosaic	5	5		
Metals	0.70	0.5	0.4	
Wood	0.51	0.3		
Glass	1	1		
Cardboard and paper	1	1		
Sack	0.50	0.3		
Total	100	100	100	100

The composition of C&D waste related to some parameters such as types of the structures, age of the structures, method of building, method of demolition, materials used in construction. For example, debris resulted in new construction is likely to contain significant amounts of plastics and drywall laminates, while older buildings may contain lead piping and plaster [10].

The composition of C&D waste consists of wood, metals, concrete, soil, stone, paper, broken bricks, mixing sand and cement, gypsum, asphalt, rock, ceramic tile, mosaic, cardboard and paper, glass and sack. Salvageable materials like wood and metals usually resold or reused that caused to reduce the cost of collection and transfer of wastes. There are some scrap dealers in Tehran that collect the salvageable materials and sold them to secondhand buyers. Unfortunately this system of collecting and sale of these materials by collectors cannot have a benefits at an efficient level, although it may consider as an effective application of reuse and recycling [22]. So, municipality’s significant attention is required to handle this situation.

Because most of C&D waste in Tehran (Fig. 2) consist of mixing sand and cement, concrete, broken bricks and soil that is similar to Kermanshah, Shahrood and Mashhad. But Tehran and Kermanshah has the most similarity of composition of C&D waste. This could be due to similarity between types of constructions were demolished. A small portion of this waste was sent to Rigsazan factory near the Abali landfill and recycled to sand. But significantly portion of them were buried.

According to previous study, the composition of C&D waste in Brazil consists of mineral C&D Waste (65%), Wood (13%), Plastics (8%) and others (14%) [23]. Another study were conducted in Norway about C&D waste composition showed that C&D waste in Norway consisted of Asbestos (0.38%), Hazardous waste (0.07%), Concrete/Bricks (67.24%), Gypsum (2.77%), Glass (0.26%), Insulation/EPS (0.49%), Metal (3.63%), Paper/Cardboard/Plastics (1.14%), Wood (14.58%) and unknown composition (9.44%) [2]. These composition shows nearly composition to Tehran C&D waste composition.

In other study in Northeast, the C&D waste composition was reported as fallow: Plastics (2%), Metals (5%), Concrete and Rubble (ABC) (9%), Roofing (11%), Drywall (10%), Wood (34%) and other waste (29%) [24, 25]. Also according to a study was conducted on the C&D waste composition in New Zealand, the C&D waste were consisted of wood fiber and timber (38%), concrete (25%), plastic board (18%), iron and other metals (6%), paper and Cardboard (3%), organic (2%), plastic (1%), glass (1%), hazardous materials (1%) and others (5%) [15]. These composition seems a little different with Tehran’s composition and it can be because of different methods and technologies of construction and demolition, difference between materials used in construction, different geological condition and different social and economic conditions.

Incuriosity to the number, amount and size of the products utilized, lack of knowledge about construction, operation and maintenance during design activities, lack of interest of contractors may also affect the waste generation and composition at a construction site [16, 26].

Other factors such as lack of attention to the safety and direction related to the materials, poor materials selection and handling which may result in loss raw materials and breaking parts are also significant. Some parts of initial construction materials (about 1 to 10% by weight) may be lost at the site and consider them as waste.

### C&D waste management in Tehran

In Tehran, C&D waste is managed and disposed with municipal solid waste (MSW). These wastes have been collected by Tehran municipality’s contractors and transferred to landfills located in the suburb of Tehran. Abali and Kahrizak are two of active landfills that C&D wastes of Tehran transferred there. Also according to some available information, the most part of C&D waste is dumped in the uncontrolled sites or other improper sites such as roadsides that can make some problem such as creating undesirable views, blocking the paths, contaminating the waste and environment and etc. Thus C&D waste may be contained a variety of different materials such as some contaminants enter to C&D wastes from outside of building and/or generation place of the construction and demolition. So, C&D waste sorting should be done for recycling of this waste [23].

Considering some laws and policies to motivate contractors and companies for transferring the waste to landfills and record the real amount of generated is so important. Although based on MAPSA data, 360 teams cruise and control the illegal disposal of MSWs in inappropriate sites, but this number of teams is inadequate and cannot be effective in controlling this problem due to the expansion of Tehran.

Results of a study in Bangkok showed that there are about 69 construction and renovation companies that usually also administered the management of its wastes and about 31 companies assigned their waste management to subcontractors or other companies [16]. Also, the results of other studies showed that the majority of C&D waste is managed as a disposal in illegal sites or other uncontrolled sites [1, 16]. Other study in Australia showed that the Australian government, similar to other countries, had legislation to decrease the landfill requirements for disposal of solid wastes by 50% until 2000. According to this program, a multinational construction company has made a company policy that manage all the solid waste generated on site and, finally conclude to decrease the quantity of wastes reach to landfill sites. Based on the results showed that total volume of waste generation was decreased by 15% at source reduction and prior to recycling and 43% less waste reach to the landfill after implementing this program. Cost saving related to waste handling charges was 50% [27].

Based on research on Germany and Australia related to the C&D waste management, minimization plays a significant role to achieve the goal of sustainability in management of the construction waste. For redacting C&D waste management activities, government has improved the statutory program and tools for minimizing waste generation and encourage all people and residents to waste recovery. The effectiveness and successful rate of these statutory frameworks may lead to create a various waste management program in different countries. However, countries can use of success experiences for improving waste management rules and activities related to waste minimization and recovery [9, 28]. Also, Brazil has a good experience and practical plan for C&D waste disposal and management. According to their experiences, the C&D waste management systems that traditionally operate, are very expensive for local authorities and have several adverse impacts for health as well as environment. Based on the results of C&D waste disposal and management obtained in Brazil, policy focus only on the transportation and landfilling program of C&D waste is not able to manage the uncontrolled and illegal dumping. The policy for C&D waste management must be completed with a transfer stations network which declined the transportation and operational costs and make the uncontrolled dumping less efficient and attractive. Despite C&D waste landfills seem a simple and feasible option special in small towns, recycling plan can use as practical tool in megacities such as Tehran [23].

The problems related to C&D waste management in megacities like Tehran is similar to other cities in Thailand and Hong Kong. Some of these problems are: 1) Insufficient funds allocated for MSW management and inappropriate method used for collection; 2) lack of effective plan for establishing disposal equipment and facilities in the abutting area; 3) lack of guidelines and/or direction for regulating the construction and demolition waste management hierarchy program from minimization and separation of source, collection, transportation, storage, controlling and monitoring and disposal; 4) insufficient skilled personnel in implementing an effective management program (special for collection and disposal); 5) no plan for waste recycling; 6) lack of efficient legislation; 7) non-public participation; and 8) lack of government legal fulfilment [16, 29, 30]. The effective implementation of waste management program in Tehran includes minimizing raw material consumed in the designing phase, recycling and/or reducing fragment or waste unused at construction place, reusing waste or unused materials. Benefits of the implementation of this program include: Protecting the environment by reduce in usage of energy and natural resources, cost saving, creating job opportunities, making a good market for waste materials. Some activities such as finding the best strategies to reduce C&D waste volumes, conducting an informal waste audit, using recyclable and reusable materials in design of products, training employers, contractors, and subcontractors, setting up an effective separation program, The practice of law by all segments of society must be done to achieve this goals [10]. In addition, government and national secondary material administrative system should support this program and public awareness can be increased [31].

Generally, for overcoming to Construction and demolition (C&D) waste management issues in Tehran as the biggest city of Iran, paying attention to reuse, minimization and recycle program of the C&D solid waste and decreasing the amount of buried waste, using new technologies in this field and the successful experiences of other countries are recommended.

### Predicted waste production in future

**Fig. 3:**
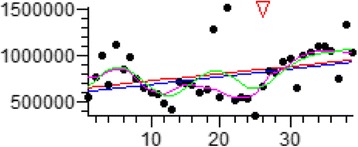
C&D waste generation in 2011–2021

Although predicting the exact amount of generated C&D waste in future cannot be easy but it can help to set the proper plan up for waste management in future. Figure 3 and equation 1 define the amount of wastes entered to the landfill:

Nonparametric regression analysis:

(Not assuming a normal distribution)

1 $$ \mathrm{Value} = \mathrm{a} + \mathrm{b}\mathrm{x} $$

x = Intended month

a = 604002.059

b = 8270.294 (95 C.I.: -350.000 to 15952.500)

Change-point test for continuous variables:

(Low *P* value indicates a significant change at some points during the sequence)

Two-tailed *P* = 0.001

The change occurs after value no. 26

According to the results, variation point occurred in 2013 that was shown in Fig. 3 by triangle mark. Also, *p* values indicate the acceptable possibility.

So, C&D waste generation in Tehran in next ten years is shown in Table 5. In this case, the coefficient for converting volume to weight ratio was 1.5. Based on the results, 2,784,158 t waste will generate in 2025 and generation rate will increase 122% in comparison with 2016 that is so considerable. In addition, predetermined plan is needed to manage properly construction and demolotio (C&D) waste management in Tehran. Prediction study of C&D waste generation in Norway was used simple and plain model for flows and material stocks. Monte Carlo simulation was developed to estimate the unreliability associated with the input data and factors. According to this simulation, the obtained results showed a notable increment in construction and demolition waste in the future for the large and huge fractions such as wood, plastic and concrete/bricks. It was concluded that these studies can make valuable and useful data source to anticipate the future requirements for handling and treatment capacity, the composition of waste and problems will meet in waste handling systems [2].

**Table 5:** Generated waste in Tehran in next 10 years (2016–2025)

Year	Volume (m^3^)	Weight (Ton)
2016	711,515	1,234,479
2017	810,758	1,406,666
2018	910,001	1,578,852
2019	1,009,244	1,751,039
2020	1,108,487	1,923,225
2021	1,207,730	2,095,412
2022	1,306,973	2,267,599
2023	1,406,216	2,439,785
2024	1,505,459	2,611,972
2025	1,604,702	2,784,158

## Conclusion

According to this results, the main results obtained from this study follow as:

- The mean of generated construction and demolition waste in Tehran was about 16,529,210 m^3^ per year and 3.2 t per capita. Most of these wastes were transferred to the landfill and others were dumped illegally in the certain sites that caused to Losing energy, money and natural resources.
- Management of C&D Wastes in Tehran are not desirable and application of innovative methods used in developed countries is essential.
- For implementation of effective waste management in Tehran, some requirements such as minimizing the material used in the design and planning phase, decreasing scrap and large fragment and other wastes at construction site, reusing and recycling materials used on site, and recycling wastes that cannot be reused on site should be considered.
- Benefits of this programs are: Protecting the environment with reduce in using energy and natural resources, cost saving, making job opportunities, creating a market for recycled or reuse waste.
- Lack of C&D waste data in Tehran and access to existing data are the main limitations in this study. Therefore, design of plan for future requirements for construction and demolition waste management is really hard and it is essential that municipal of Tehran creates a statistical database about C&D waste, determining the exact amount of generated waste in place, the number of construction and demolition license and contractors that worked in this field. Finally, an effective C&D waste management program could be designed according to these data.
